# Comparison of Spaceborne and UAV-Borne Remote Sensing Spectral Data for Estimating Monsoon Crop Vegetation Parameters

**DOI:** 10.3390/s21082886

**Published:** 2021-04-20

**Authors:** Jayan Wijesingha, Supriya Dayananda, Michael Wachendorf, Thomas Astor

**Affiliations:** Grassland Science and Renewable Plant Resources, Universität Kassel, Steinstraße 19, D-37213 Witzenhausen, Germany; supriyad24@gmail.com (S.D.); mwach@uni-kassel.de (M.W.); thastor@uni-kassel.de (T.A.)

**Keywords:** monsoon crops, leaf area index, leaf chlorophyll concentration, crop water content, multispectral, hyperspectral

## Abstract

Various remote sensing data have been successfully applied to monitor crop vegetation parameters for different crop types. Those successful applications mostly focused on one sensor system or a single crop type. This study compares how two different sensor data (spaceborne multispectral vs unmanned aerial vehicle borne hyperspectral) can estimate crop vegetation parameters from three monsoon crops in tropical regions: finger millet, maize, and lablab. The study was conducted in two experimental field layouts (irrigated and rainfed) in Bengaluru, India, over the primary agricultural season in 2018. Each experiment contained *n* = 4 replicates of three crops with three different nitrogen fertiliser treatments. Two regression algorithms were employed to estimate three crop vegetation parameters: leaf area index, leaf chlorophyll concentration, and canopy water content. Overall, no clear pattern emerged of whether multispectral or hyperspectral data is superior for crop vegetation parameter estimation: hyperspectral data showed better estimation accuracy for finger millet vegetation parameters, while multispectral data indicated better results for maize and lablab vegetation parameter estimation. This study’s outcome revealed the potential of two remote sensing platforms and spectral data for monitoring monsoon crops also provide insight for future studies in selecting the optimal remote sensing spectral data for monsoon crop parameter estimation.

## 1. Introduction

The global cropland area is predicted to decline by 1.8–2.4% by 2030 due to conversion of arable croplands to mostly built-up landcover, and 80% of this land cover change is expected to occur in Asia and Africa [1]. Bengaluru is one of the megacities (over 10 million population) in southern India [2], which has already lost 62% of the vegetated area, while the urban area increased by 125% between 2001 and 2011 [3]. Agricultural production has intensified (i.e., high nitrogen (N) fertiliser usage, drip irrigation), and the cropping pattern has changed to meet the increasing food demand for the growing population. Between 2006 and 2012, the cropping pattern in Bengaluru changed from high water use paddy cultivation to dry land cereals and pulses (e.g., maize, finger millet, lablab). According to the state-level statistics, maize and finger millet crop yield increased by 4 to 6% annually, while pulse yield (including lablab) soared by 15% [4].

Increasing crop production using available arable lands while sustainably managing resources (e.g., water, soil) and reducing climate change is challenging [5]. Thus, near-real-time crop status monitoring could be a way forward to manage available resources and reduce inputs (i.e., precision agriculture). However, crop monitoring approaches need to be adapted to distinct crop types, in different growth stages (phenology), and under different agricultural practices. Remote sensing (RS) is one of the primary tools for crop monitoring [6]. RS facilitates contactless data collection over a given crop area using reflected electromagnetic energy, enabling the characterisation of an area′s spatiotemporal information. The development of RS data collection and analysis techniques helps to achieve accurate models to estimate crop parameters.

Various sensor platforms (i.e., terrestrial, airborne, and spaceborne) have been employed to collect data about cropping areas and estimate crop growth and health parameters through different modelling approaches [7]. Generally, the reflected electromagnetic energy from the plant changes according to the physiological and the structural condition of crops and the surrounding environment [8]. Both multi- and hyperspectral sensors have been utilised from different platforms to capture these varying reflected energies. Hyperspectral sensors capture reflected energy at many narrow spectral bands (usually more than 30 bands). In comparison, multispectral sensor data contains fewer spectral bands with larger bandwidth [9]. Due to the higher spectral sensitivity of the hyperspectral data, there is a significant potential to capture a wider variety of different physiological and structural crop traits [8]. To make the clear comparison of the spectral resolution difference of the RS data for crop trait estimation, it is necessary to obtain RS data with similar spatial resolution. However, most studies which compared the spectral resolution sensitivity (hyperspectral vs. multispectral) for crop trait estimation were based on different spatial resolution; for example [10] employed field spectroscopy data as hyperspectral data with point observation and satellite data as multispectral data with 10 m spatial resolution for estimation of maize crop traits.

Empirical (statistical) models (both parametric and non-parametric) or physical models (e.g., radiative transfer model inversion) have been employed to estimate crop parameters using spectral data [11]. The empirical models inspect the association between in-situ measured target crop vegetation parameter and spectral reflectance data collected from RS. The reflectance data or their transformations (e.g., first derivative) or vegetation index (VI) developed from many wavebands were the inputs for the empirical models. A linear regression model is one of the standard parametric empirical modelling methods which estimates crop traits by utilising single waveband reflectance data or VI data as input [12]. In contrast, all—or only the essential—waveband reflectance data (original and transformed) and a multitude of VI data can be used as inputs for non-parametric empirical modelling with, e.g., machine learning methods (i.e., random forest, Gaussian process) [13]. Since both parametric and non-parametric models are data driven methods, a comparison of these methods for estimation of crop traits using RS spectral data can always provide capabilities of different modelling methods [14].

Many crop vegetation parameters that indicate growth and health status have been estimated using RS spectral data, e.g., leaf area index (LAI), leaf chlorophyll content (LCC), and canopy water content (CWC) [7]. LAI (m^2^/m^2^) is the leaf area per unit ground area, an essential plant biophysical variable to understand growth, health, and yield [15]. When considering other photosynthetically-active plant parts besides the leaves, it is called the green area index or plant area index [8]. Crop LAI estimation using RS reflectance data and empirical modelling approaches (both parametric and non-parametric) have shown promising results, but also considerable variation in prediction quality (coefficient of determination (R^2^) ranges from 0.36 to 0.97) [16].

The LCC (both chlorophyll a and b) is a crop biochemical indicator for photosynthetic capacity, environmental stress, and N status of leaves [17,18]. LCC (µg/cm^2^) is referred to as leaf-level quantification, while the multiplication of LCC with LAI is considered canopy chlorophyll content (CCC-g/cm^2^). Spectral reflectance from the green to near-infrared region shows a strong relationship with LCC values [8]. According to available literature, LCC can be estimated with a maximum relative error of less than 20 % from both multi- and hyperspectral sensors [19,20].

Quantification of CWC (g/m^2^) attempts to identify crop water stress by estimating the quantity of water per unit area of the ground surface [21]. Water absorption regions (970 nm and 1200 nm) of the spectral reflectance data have been employed to estimate CWC using RS spectral data [21,22,23]. However, few studies were able to accurately estimate (R^2^ > 0.7) maize crop CWC using linear regression models with VI derived from wavebands from the green, red-edge and near-infrared regions [24,25]. Conversely, the crop CWC has not yet been estimated using full spectral data to uncover the full potential of hyperspectral information.

Successful estimation of crop vegetation parameters with RS spectral data has been demonstrated for various crop types such as wheat, rice, barley, and maize [7,26,27]. However, RS data application has not been examined for crops like finger millet and lablab, which are major monsoon crops in the tropical region (e.g., Bengaluru, Southern India). Furthermore, few studies have compared different remote sensing platforms (e.g., in-situ vs airborne vs spaceborne) and sensors (multispectral vs hyperspectral) for crop vegetation parameters estimation [16,28]. Thus, this study sought to fill the identified research and knowledge gap for RS for monsoon crop monitoring. The primary objective of this study is to evaluate two different RS spectral data types (420–970 nm) with a similar spatial resolution (~1 m), namely spaceborne multispectral (WorldView3–8 bands) and unmanned aerial vehicle (UAV) borne hyperspectral (Cubert–126 bands) for estimating three crop vegetation parameters (LAI, LCC, and CWC) from three crop types (finger millet, maize, and lablab) under different agricultural treatments (irrigation and fertiliser). The specific sub-objectives of this study were:

- To build crop-specific parametric and non-parametric models to estimate crop vegetation parameters
- To evaluate the developed vegetation parameter estimation models against (a) the spectral sensitivity of the RS data (multispectral vs hyperspectral), (b) modelling method (parametric and non-parametric), and (c) crop type (finger millet, maize, and lablab)
- To explore how crop-wise vegetation parameter estimation is affected by agricultural treatment (irrigation and fertiliser)

## 2. Materials and Methods

### 2.1. Study Site and Experimental Design

This study was performed in an experimental station on the premises of the University of Agricultural Science (UAS), Bengaluru, Karnataka state, India (12°58′20.79″ N, 77°34′50.31″ E, 920 m.a.s.l). The climate of the study area is a tropical savanna climate with 29.2 °C mean annual temperature. The south-west monsoon rain between June to October contributes substantially to the mean total annual rainfall of 923 mm. The dominant soil types in the area are Kandic Paleustalfs and Dystric Nitisols.

**Figure 1:**
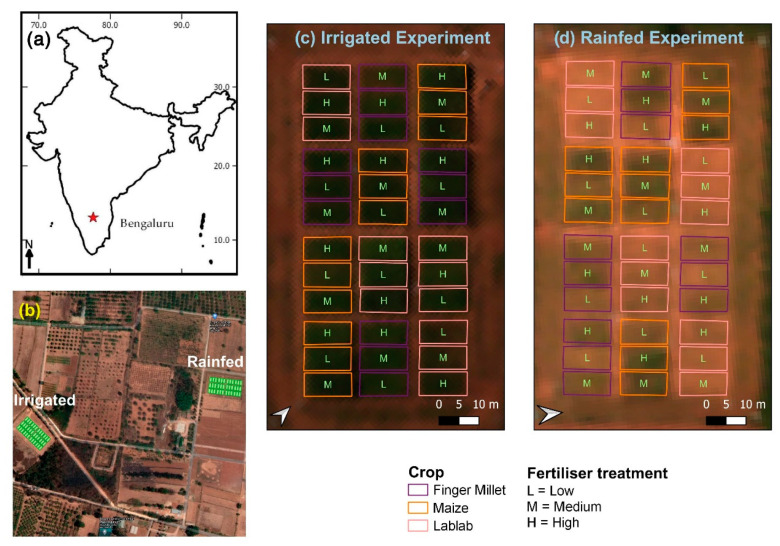
(**a**) Bengaluru, India; (**b**) Overview of the two experiment sites overlaid with Google satellite layer; (**c**) irrigated experiment layout, and (**d**) rainfed experiment layout with true colour composite Cubert hyperspectral image (Red = 642 nm, Green = 550 nm, Blue = 494 nm).

Two experimental layouts were established with two water treatments: drip irrigated (I) (controlled according to available precipitation), and rainfed (R) (Figure 1). The experiment was conducted in the 2018 Kharif season (July–October). In each experimental layout, four repetitions of finger millet (*Eleusine coracana* L.) (cultivar ML-365), maize (*Zea mays* L.) (cultivar NAH1137), and lablab (*Lablab purpureus* L.) (cultivar HA3) were cultivated with three different N fertiliser treatments (low, medium, and high) (36 blocks within one experimental layout). At the high fertiliser level, the recommended dosage of N fertiliser (50 kg N ha^−1^, 150 kg N ha^−1^, and 25 kg N ha^−1^, respectively, for finger millet, maize and lablab) was applied. A reduced amount was applied at medium fertiliser treatment (58%, 56%, and 53% of the recommended N dosage, respectively, for finger millet, maize, and lablab). No N fertiliser was applied in the low-level fertiliser treatment for the three crop types. Phosphorous (P) and potassium (K) fertiliser were applied at the time of sowing at different levels following the recommended doses for the respective crop types [29].

A single crop block was 6 m by 12 m, and the crop blocks were designed in a randomised block design. Each block was divided into two parts for destructive sampling (i.e., CWC) and non-destructive sampling (i.e., LAI, LCC). Field-level data collection and RS data collection campaigns were conducted between 29–31 October 2018. The phenological stages of the crops at the time of the field campaign are summarised in Table 1.

### 2.2. In-Situ Field Data

Block-level LAI and LCC data were collected as non-destructive measurements. LAI was measured using an LI-COR LAI-2000 plant canopy analyser (LI-COR Inc., Lincoln, NE, USA). One single LAI measurement consisted of a three-time repetition of one above-canopy measurement followed by four below-canopy measurements between two crop rows. [30]. All LAI measurements were performed after 16:00 when the sun was at the horizon. LCC was measured using a handheld SPAD-502 Plus Chlorophyll meter (Konica Minolta, Osaka, Japan). The device measures the absorbances of the leaf in red and near-infrared regions. The device retrieves an arbitrary, unitless, numerical ‘SPAD’ value (SV) based on absorbance values. Four plants were randomly selected in each block, and three measurements per plant from the last fully developed leaf were taken. The block-level SV was computed as the average of all 12 measured SVs. According to [31], the consensus regression Equation (1) was applied to convert the SV into LCC in µg/cm^2^:

(1) $$LCC\;\left({\mu g\;\mathrm{cm}}^{-2}\right)=\frac{\left(99\times SV\right)}{\left(144-SV\right)}$$

After LAI and LCC measurements, destructive biomass sampling was conducted. From each block, two plants were removed, and above-ground fresh biomass weight was recorded. A subsample was dried using a sun dryer (maximum temperature was 75 °C) until no further weight loss was found (approx. 3 days). Based on dried sample weight, total dry biomass weight was computed. According to the sampled plant area, fresh biomass content (kg/m^2^) and dry biomass content (kg/m^2^) were determined. The canopy water content (CWC) was computed (Equation (2)) using fresh and dry biomass contents [22]:

(2) $$CWC\;\left({\mathrm{kg}\;m}^{-2}\right)=fresh\;biomass\;content-dry\;biomass\;content$$

### 2.3. Remote Sensing Data

RS datasets acquired from two platforms and sensor systems were utilised in this study: (a) multispectral WorldView3 satellite data, and (b) hyperspectral Cubert UHD data mounted on a UAV.

#### 2.3.1. WorldView 3 Data

A WorldView-3 multispectral satellite scene from 26 October 2018 was used as satellite RS data. The satellite image contained eight multispectral bands between 397 nm to 1039 nm, covering the visible and near-infrared regions of the electromagnetic spectrum (Table 2). The image′s spatial resolution is 1.24 m [32,33].

The fast line-of-sight atmospheric analysis of spectral hypercubes (FLAASH) method in ENVI 5.0 software (Harris Geospatial Solutions Inc., Broomfield, CO, USA) was applied to pre-process the satellite image using the image′s metadata [34]. The pre-processed image pixel contained atmospherically-corrected surface reflectance values. However, the coastal blue (CB) band from WorldView3 data was not incorporated for the crop parameter vegetation modelling due to substantial influence from atmospheric scattering. Additionally, six vegetation indices (VIs) were calculated (Table 3). These VIs were chosen from published literature due to their proven potential to estimate LAI, LCC, and CWC [12,23,35] and compatibility with WorldView3 wavebands.

#### 2.3.2. Cubert Hyperspectral Data

A custom-made octocopter equipped with the Cubert Hyperspectral FireFleye S185 SE (Cubert GmbH, Ulm, Germany) snapshot camera was utilised as a UAV-borne imaging system. The hyperspectral camera is a 2D imager with a multi-point spectrometer. The camera has 450–998 nm spectral sensitivity and contains 138 spectral bands with a 4-nm sampling interval. The bands′ full width at half maximum value is 4.8 nm at 450 nm and 25.6 nm at 850 nm. The spectral image is 50 by 50 pixels in size, and the camera focal length is 12 mm. Additionally, the camera has a panchromatic sensor that provides images with 1000 by 990 pixels [41,42].

The UAV-borne hyperspectral images were acquired on 29–30 October 2018 in both irrigated and rainfed experimental sites between 11:30–14:00 under clear sky conditions. At each site, the UAV-borne dataset was collected at 100-m flying height. According to the flying height, the ground sampling distance of the UAV dataset was 1.0 m. All flight missions were configured to keep 80% overlap (forward and side), and the UAV was flown with 2 ms^−1^ horizontal speed. Before each UAV flight, the camera was radiometrically calibrated to obtain surface reflectance values using a white calibration panel [43,44]. For georeferencing, the UAV images, 1-m^2^ ground control points (black and white wooden crosses) were laid on the ground before the flights, and the positions of points were measured using a Trimble global navigation satellite system.

A workflow described by [43] was applied to produce a digital ortho-mosaic from single UAV-borne hyperspectral images using Agisoft PhotoScan Professional version 1.4.1 (64 bit) software (Agisoft LLC, St. Petersburg, Russia). Due to noise in the spectral bands between 450–470 nm, the final ortho-mosaic contained only 126 spectral bands (470–970 nm). Six VI images were computed in addition to the spectral band images (Table 3).

### 2.4. Model-Building Workflow for Crop Vegetation Parameter Estimation

From the WorldView-3 satellite dataset (WV3) and the UAV-borne hyperspectral dataset (CUB), mean values were extracted from the non-destructively sampled portions of the plots for (a) vegetation indices (VIs), and (b) all spectral wavebands (WBs). A 2-m internal buffer to the plot was applied to avoid edge effects. To estimate the crop parameters (LAI, LCC, and FMY) for each crop type, (a) parametric modelling (linear regression-LR) was conducted using VIs, and (b) non-parametric modelling (random forest regression-RFR) was performed with selected WBs based on feature importance analysis.

The relationship between the estimator (e.g., VI) and the dependent variable (e.g., LAI) was built using a linear equation (straight line) in the LR models. Before the LR model was built, a crop-wise Pearson correlation coefficient (r) was computed between the crop vegetation parameter and the VIs. A single LR model using the highest correlated VI was built to estimate crop-wise vegetation parameters.

RFR is one of the most prominent non-parametric regression algorithms that has been frequently applied for crop parameter modelling with RS data [13]. It is an ensemble modelling approach that employs decision trees and bagging [45]. This ensemble tree-based architecture supports the handling of a multitude of correlated variables [46]. The most influential bands were identified using the Boruta feature selection algorithm to reduce the computational intensity and overfitting. Boruta is an iterative process: in each iteration, features with a lower contribution to the accuracy were removed, and new random variables were introduced, thereby selecting essential variables for the model [47]. From the Boruta feature selection method, specific WBs from CUB and WV3 data were selected for crop-wise vegetation parameters. The selected WBs were utilised to build RFR models. Based on [48], one-third of the number of estimators was set as ′the number of drawn candidate variables in each split-(*mtry*)′ hyperparameter value in each RFR model. The other hyperparameter, ‘the number of trees in the forest’ and ‘the minimum number of observations in a terminal node–(*node size*)’, were kept as 500 and 5, respectively, for all RFR models.

Additionally, the importance of the selected wavelengths was determined using the actual impurity reduction (AIR) importance value [49]. The AIR is a Gini importance value that was corrected for bias. Based on AIR values, the most important waveband for estimating each crop-wise vegetation parameter could be identified.

All the modelling procedures were executed using the ‘mlr3′ library and its extensions in the R programming language [50,51]. The ‘ranger’ library was employed inside the ‘mlr3′ library to build RFR models [52], and the ‘Boruta’ library was utilised for the feature selection step [47]. In total, 12 models for each crop vegetation parameter were developed (i.e., 2 modelling methods [LR and RFR] × 2 RS datasets [CUB and WV3] × 3 crops [FM, MZ, and LB]).

Due to limited data (24 data records per crop), cross-validation (CV) was applied in the model-building workflow. In CV, 12 models were trained and validated as follows: one data point from the irrigated site and one data point from the rainfed site were left out each time for validation, and the remaining 22 points (11 from the irrigated site and 11 from the rainfed site) were utilised for training the model. Based on the predicted vs actual values in the validation phase, the root means squared error (RMSE) was computed Equation (3). To standardise the RMSE values, normalised RMSE (nRMSE) was calculated by dividing RMSE from the range of the corresponding crop parameter value (the difference between the minimum and maximum values) Equation (4). The coefficient of determination (R^2^) [53] was computed based on actual and predicted values Equation (5). Based on the distribution of the nRMSE and R^2^ values, the crop-wise best model was identified for each crop vegetation parameter. Moreover, each model′s predictive capability was examined using normalised residual values Equation (6) against two water and three fertiliser treatments. Positive or negative normalised residual values indicate overestimated or underestimated values, respectively:

(3) $$RMSE=\sqrt{\frac{1}{n}\overset{n}{\underset{i=1}{\sum }}{\left(y_{i}-{\hat{y}}_{i}\right)}^{2}}$$

(4) $$nRMSE=\frac{RMSE}{\left(max\left(y\right)-min\left(y\right)\right)}\times 100$$

(5) $$R^{2}=\left[1-\frac{\sum_{i=1}^{n}{\left(y_{i}-{\hat{y}}_{i}\right)}^{2}}{\sum_{i=1}^{n}{\left(y_{i}-{\bar{y}}_{i}\right)}^{2}}\right]$$

(6) $$normalised\;residual\;value=\frac{\hat{y}-y}{\hat{y}+y}$$

where y is the actual crop vegetation parameter, $\hat{y}$ is the predicted parameter, $\bar{y}$ is the average value of the actual parameter, and n is the number of samples.

## 3. Results

### 3.1. Crop Vegetation Parameter Data

The descriptive statistics of the crop-wise parameter data are presented in Table A1. The LAI values ranged between 0.4–3.2 m^2^/m^2^ for finger millet, 0.2–3.5 m^2^/m^2^ for lablab, and 1.0–3.0 m^2^/m^2^ for maize in both the irrigated and rainfed sites. For all three crops, the irrigated field always showed considerably higher LAI values than the rainfed site. According to the crop-wise two-way analysis of variance (ANOVA) test for LAI values, significant differences (*p* < 0.001) in LAI between irrigation treatments (I and R) were found for all three crops. N fertiliser (low, medium, and high) did not significantly affect (*p* > 0.1) LAI for any of the crops. However, there was a significant effect of interaction between irrigation and N fertiliser for lablab LAI (*p* = 0.03), with the combinations of N fertiliser and irrigation increasing average LAI.

The highest average LCC was found in irrigated maize (76.4 µg/cm^2^), while the rainfed finger millet had the lowest average LCC (10.2 µg/cm^2^) (Table A1). Like LAI, irrigation significantly positively affected (*p* < 0.001) LCC for all three crops. Fertilizer only significantly affected maize LCC (*p* = 0.03) positively. In contrast, there was a significant effect from the interaction between irrigation and N fertiliser for both finger millet (*p* = 0.01) and maize (*p* = 0.05) for LCC, with N fertiliser combinations irrigation increasing.

The highest CWC was found for maize (average CWC = 1.5 and 0.9 kg/m^2^ for irrigated and rainfed) (Table A1), whereas lablab had the lowest CWC (0.7 kg/m^2^ and 0.08 kg/m^2^ for irrigated and rainfed experiments, respectively). According to the ANOVA test, CWC was significantly affected by irrigation for finger millet, lablab, and maize (*p* < 0.001). Besides, the CWC for finger millet revealed a significant positive effect of fertiliser (*p* = 0.05) and the interaction between water and fertiliser (*p* = 0.01).

Crop-wise LAI was strongly correlated with CWC (r = 0.85, 0.78, and 0.74 for finger millet, maize, and lablab, respectively). Similarly, crop-wise LCC was also positively correlated with both LAI (r = 0.81, 0.64, and 0.60) and CWC (r = 0.60, 0.62 and 0.54) for finger millet, maize, and lablab, respectively.

### 3.2. Spectral and Vegetation Index Data

**Figure 2:**
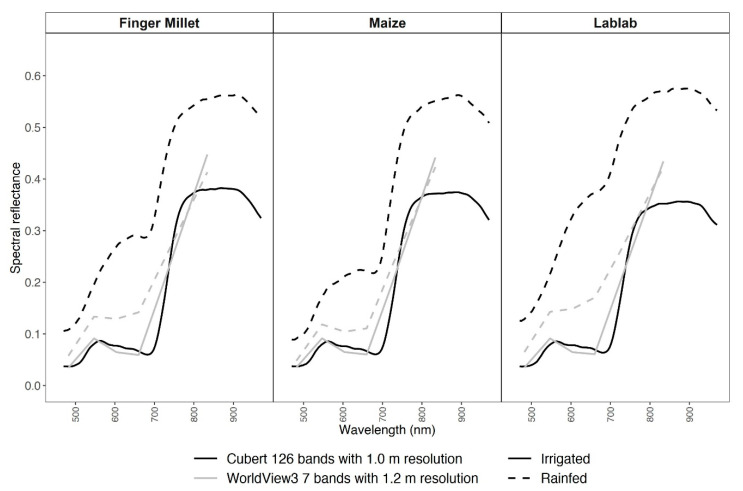
Average spectral reflectance data for millet, lablab, and maize from Cubert (black) and WorldView3 (grey) data for irrigated (solid line) and rainfed (dashed line) experiments.

The pattern of the spectral reflectance curves from the two experimental sites (I and R) exhibited a substantial difference for both RS datasets (Figure 2). In the irrigated plots, both CUB and WV3 spectral curves followed a typical healthy vegetation spectral reflectance curve. However, in the rainfed data, both CUB and WV3 reflectance data deviated in the visible region of the spectrum from healthy vegetation spectral curve due to higher soil spectral signals (Figure 1c).

**Figure 3:**
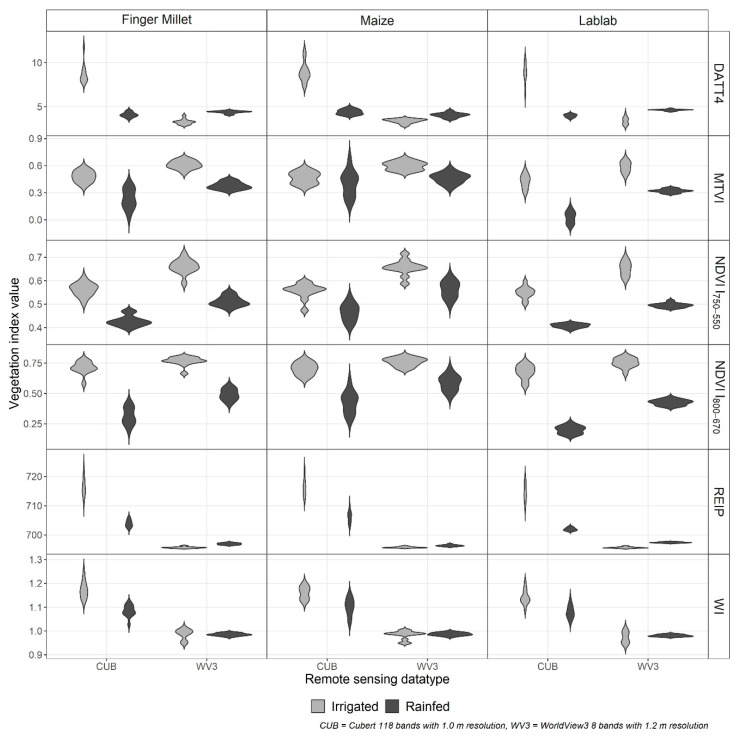
Distribution of crop-wise vegetation indices (VI) for finger millet, lablab, and maize from Cubert (CUB) and WorldView3 (WV3) data from irrigated (grey) and rainfed (black) experiments. (NDVI: normalised difference vegetation index, DATT4: The 4th VI introduced by Datt (1998), MTVI: modified triangular vegetation index, REIP: red-edge inflexion point, and WI: water index).

The crop-wise VI significantly differed (*p* < 0.001) between the two RS data types (CUB and WV3) as well as between the irrigation treatments (Figure 3). However, the WV3 water index (WI) was the only index that did not show a substantial difference (*p* > 0.3) between the irrigation treatments.

### 3.3. Crop Vegetation Parameter Estimation with Linear Regression

LR models with VI were employed to estimate crop-wise vegetation parameters using two RS datasets. A total of six models were built separately (3 crops × 2 RS datasets). LAI estimation from CUB and WV3 data showed similar results for all three crops (Table 4). All six models obtained R^2^_cv_ ≥ 0.73. CUB VI for LAI estimation achieved nRMSE_cv_ of 15.7 %, 14.9 %, and 15.6 % for finger millet, maize, and lablab, respectively (Table 5). Likewise, nRMSE_cv_ of 16.1 %, 15.9 %, and 16.0 % were obtained for finger millet, maize, and lablab LAI estimation, respectively, using WV3 data. NDVI_800_670_ was the best VI for LAI estimation using CUB data for all three crops. For WV3 data, NDVI_800_670_ was the best for maize LAI estimation, while REIP was the best VI for finger millet and lablab LAI.

The VI-based LR models for estimating LCC showed lower R^2^_cv_ values (Table 4). For finger millet, LCC estimation models with CUB VI data (nRMSE_cv_ = 18.0%) performed better than with WV3 VI data (nRMSE_cv_ = 21.0%). Maize LCC estimation models resulted in the highest normalised error and the lowest R^2^_cv_ values. From the two RS datasets, WV3 VI performed better than CUB VI for maize LCC estimation. In contrast, lablab LCC estimation models from both RS datasets showed similar performances (nRMSE_cv_ = 23.3% and R^2^_cv_ = 0.37). Of the tested VIs, NDVI (NDVI_750_550_, NDVI_800_670_) and DATT4 were the most highly correlated with LCC for both RS datasets.

Crop-wise CWC estimation from VI from two RS datasets obtained less than 20% nRMSE_cv_ (Table 4). The nRMSE_cv_ values for CWC estimation with CUB data were 19.5%, 19.9%, and 16.3% for finger millet, maize, and lablab, respectively, while nRMSE_cv_ values for finger millet, maize, and lablab were 19.9%, 17.0%, and 15.6%, respectively, for CWC estimation with WV3 data. The NDVI indices from CUB resulted in the best CWC estimation for all three crop types, while WV3-based NDVI_750_550_, REIP, and DATT4 were strongly correlated with CWC values, respectively, from finger millet, lablab, and maize.

### 3.4. Crop Vegetation Parameter Estimation with Random Forest Regression

#### 3.4.1. Key Wavebands

Important WBs for crop vegetation parameter estimation were identified using Boruta feature selection algorithms. Table 5 summarises the identified WBs from each RS datasets (CUB or WV3) for each crop vegetation parameter.

#### 3.4.2. Model Performance

RFR models were built to estimate crop-wise vegetation parameters using the identified best WBs. Irrespective of the RS datasets and crop type, the RFR models for LAI estimation yielded less than 16.1% nRMSE_cv_ and over 0.70 R^2^_cv_ (Table 6). 

The LAI estimation for lablab resulted in the lowest error among the three crop types (nRMSE_cv_ =12.9% and 12.0%, respectively, from CUB and WV3 data). The LAI estimation models for finger millet showed better performance for CUB data (nRMSE_cv_ = 16.1%) compare to WV3 data (nRMSE_cv_ = 17.1%). In contrast, CUB data and WV3 data had similar accuracy for maize LAI estimation (nRMSE_cv_ = 13.9%).

LCC estimation based on CUB data was more accurate than WV3 data for maize and lablab. For finger millet, the opposite was found (Table 6). The nRMSE_cv_ for LCC estimation with RFR was above 20.5% for all crops, regardless of the RS datatype. The nRMSE_cv_ values for LCC estimation from CUB data were 22.1%, 25.8% and 24.9%, and from WV3 data were 20.8%, 27.4 and 31.5 %, respectively, for finger millet, lablab, and maize. Based on the nRMSE_cv_, the RFR models were less accurate than the LR models for LCC estimation irrespective of the RS data type and crop type.

The R^2^_cv_ was less than 0.5 for CWC estimation for all three crops (Table 6). For finger millet (nRMSE_cv_ = 19.9%) and lablab (nRMSE_cv_ = 16.9%), CWC estimation with CUB data performed better than models with WV3 data (nRMSE_cv_ = 22.9% and 18.2%, respectively). Both RS datasets showed similar performance for maize CWC estimation (nRMSE_cv_ = 21.4%).

### 3.5. Best Models and Distribution of Residuals

**Figure 4:**
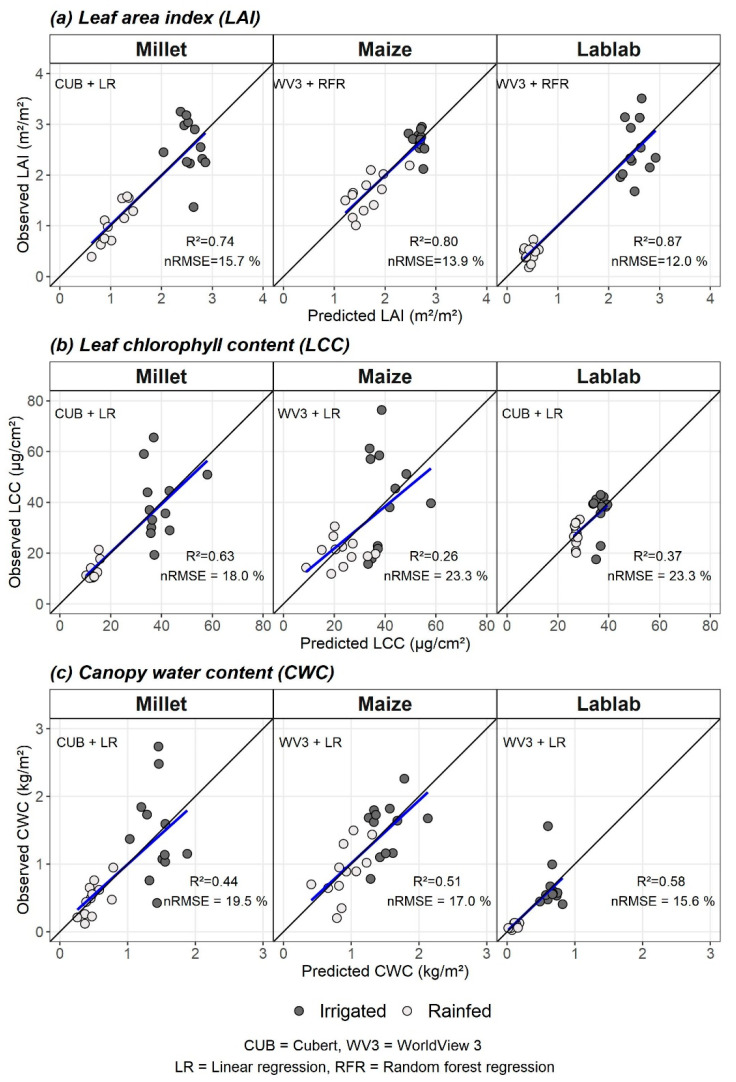
Observed vs predicted values of the best performing models for (**a**) leaf area index (LAI), (**b**) leaf chlorophyll content (LCC), and (**c**) canopy water content (CWC). The remote sensing data type (CUB or WV3) and modelling method (LR or RFR) for the best models are indicated as “RS data type + modelling method” (e.g., CUB + LR). The blue line is the fitted regression line between predicted and observed values, and the black line is the 1:1 line.

The best models from two RS datasets (CUB vs. WV3) and two modelling methods (LR vs. RFR) for each crop vegetation parameter were identified based on nRMSE_cv_. Observed vs predicted values for crop-wise vegetation parameters from the best models are plotted in Figure 4.

The normalised residual distribution values against irrigation and fertiliser treatments are shown in Figure A3. The normalised residuals of LAI, LCC, and CWC were not significantly affected (*p* > 0.05) by irrigation for any of the crops. In comparison, only the residuals from finger millet LAI and CWC prediction were significantly affected by fertiliser, with residuals decreasing from low to medium to high N fertiliser treatments.

## 4. Discussion

The main objective of this study was to evaluate two different spectral RS datasets (multispectral WV3 and hyperspectral CUB) for estimating three crop vegetation parameters (LAI, LCC, and CWC) of three major tropical crop types (finger millet, maize, and lablab). Considering the modelling method, out of the best nine (three vegetation parameters × three crop types) LR models based on VIs, CUB data provided six of the best models, while WV3 data provided three of the best models (Table 4). In contrast, out of the best nine RFR models with selected WBs, five of the best models were based on CUB data, whereas the other four relied on WV3 data. Overall, these results did not show a definite pattern between the RS datasets and the vegetation parameter estimation model′s accuracy. Similarly, [10] reported that maize LAI estimation accuracy did not significantly differ between data with two different spectral resolutions and two different modelling methods (LR vs machine learning regression). In contrast, [54] detailed that narrow band VIs derived from hyperspectral data models yielded 20% higher R^2^ values than multispectral data models for wheat and barley LAI estimation.

### 4.1. Finger Millet Vegetation Parameter Estimation

According to the authors′ knowledge, only a few studies have utilised RS data to estimate crop vegetation parameters of finger millet and lablab [29,55]. Finger millet is a small-grained cereal (C4 type) with similar crop characteristics as pearl millet, sorghum, and foxtail millet [56]. This study revealed that the hyperspectral CUB data clearly showed the substantial potential to estimate finger millet vegetation parameters irrespective of the modelling method. For finger millet LAI estimation, NDVI_800_670_ from CUB data showed the minimum error, which confirmed that NDVI has a closer relationship with LAI at lower LAI values (less than 3.2 m^2^/m^2^) [8]. Similar to these results, NDVI showed the best estimation accuracy for sorghum LAI than other VIs (i.e., greenNDVI, EVI, and MTVI2) [57].

**Figure A1:**
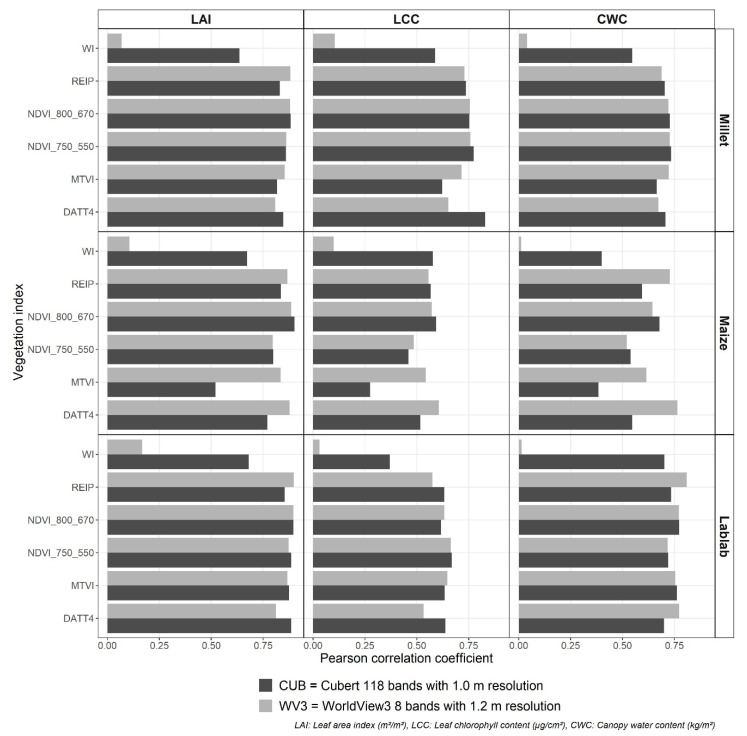
Correlation between vegetation indexes from two remote sensing data Cubert (black) and WorldView3 (grey) and crop vegetation parameters leaf area index (LAI), leaf chlorophyll content (LCC), and crop water content (CWC) for finger millet, maize, and lablab.

DATT4 is a VI for leaf chlorophyll a and chlorophyll a+b content estimation [36] and, when derived from CUB data, showed the strongest correlation with finger millet LCC (Table 4). However, DATT4 from WV3 was the least correlated VI (Figure A1). The central wavelengths of the WV3 bands do not match with the exact wavelengths of the DATT4′s formula, which may have reduced the sensitivity of the index. In contrast, Two NDVIs (NDVI_800_670_ and NDVI_750_550_) from CUB and WV3 data also showed a strong correlation with finger millet LCC (Figure A1). However, sorghum′s LCC showed the highest correlation with hyperspectral data NDVI [58] and indicated a lower correlation with multispectral data NDVI [59].

Models with VIs showed better finger millet CWC estimation results for both RS datasets. NDVI_750_550_ was the best correlated VI from both datasets, which predicts CWC indirectly [25] and contained green and near-infrared bands. CWC estimation with VI derived from green and near-infrared bands (${\mathrm{CI}}_{\mathrm{green}}=\left(\rho_{750}{/\rho }_{550}\right)-1$) also showed the best results among other VIs that predict CWC indirectly (i.e., NDVI, NDVIrededge, and CIrededge) [24]. When it comes to RFR modelling with selected WBs, WBs above 750 nm were not selected for finger millet CWC estimation. Nevertheless, some of the identified vital WBs were comparable with important WBs for finger millet fresh biomass estimation using multi-temporal terrestrial CUB data (e.g., 694 nm) [29].

### 4.2. Lablab Vegetation Parameter Estimation

Lablab is a legume crop similar to pea, beans, and lentils [60]. The lablab LAI values showed a strong correlation with NDVI values, but the LAI estimation error with NDVI was higher than the error from RFR models with selected WBs. The higher LAI values (>3.0) from lablab may impede accurately estimating LAI with NDVI due to the saturation effect, which also demonstrated by [39] with pea LAI values. In comparison to lablab LAI estimation, LR models with VI showed improved results for lablab LCC estimation. NDVI_750_550_, which contains the green band with the near-infrared band instead of the red band, was the most highly correlated VI with lablab LCC. NDVI_750_550_ is also known as ‘Green NDVI’, and according to [38], shows a strong relationship with Chlorophyll a.

**Figure A2:**
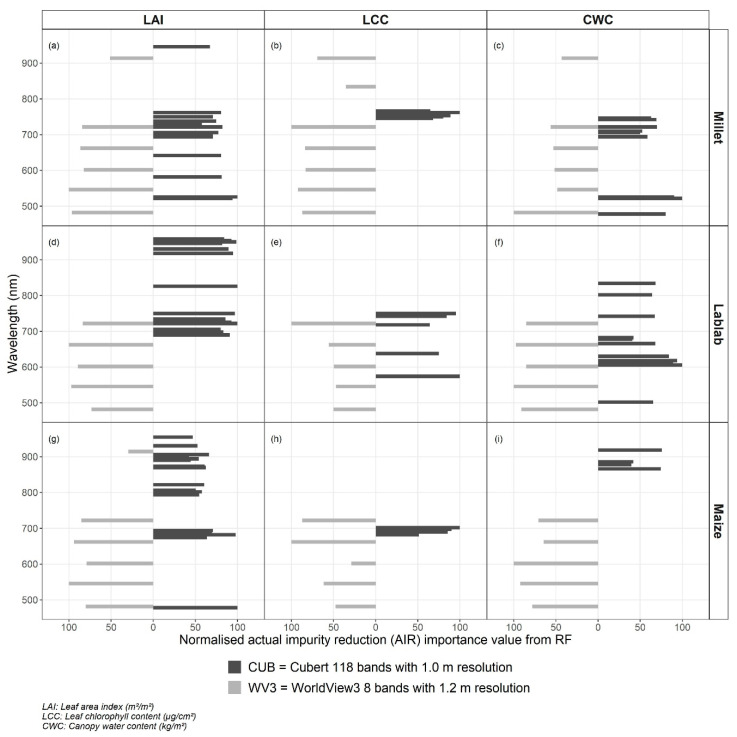
Distribution of actual impurity reduction value-based important wavebands for two remote sensing datasets (Cubert–black and WorldView3–grey) for leaf area index (LAI) estimation (**a**,**d**,**g**), leaf chlorophyll content (LCC) estimation (**b**,**e**,**h**), canopy water content (CWC) estimation (**c**,**f**,**i**) for finger millet (**a**,**b**,**c**), lablab (**d**,**e**,**f**), and maize (**g**,**h**,**i**) crops.

NDVI and REIP, respectively, from CUB and WV3 data, delivered the lowest error for lablab CWC estimation. Even though these VI do not directly relate to the leaf water content, they could determine CWC because they are linked to crop biomass [25]. Furthermore, the identified best WBs from CUB data for lablab CWC estimation (Table 5 and Figure A2) were similar to the critical WBs for lablab fresh biomass estimation [29].

### 4.3. Maize Crop Vegetation Parameter Estimation

As opposed to finger millet and lablab, maize has been frequently explored with RS data for its vegetation parameter estimation. LR modelling with hyperspectral (CUB) data to calculate NDVI showed a lower error than NDVI from multispectral (WV3) data for maize LAI estimation. [10] also revealed the same pattern for maize LAI estimation using VI from hyperspectral (field spectrometer) and multispectral (Sentinel-2) data. RFR models with essential WBs showed similar relative errors for maize LAI estimation using both RS datasets. Likewise, maize LAI estimation models from hyperspectral data and multispectral data also demonstrated similar cross-validation error (nRMSE_cv_ = 14.9 %) with a support vector machine algorithm [10].

VI derived from green, red-edge, and near-infrared bands were usually better for LCC estimation [61,62] Logically, VI containing those bands (i.e., NDIV_800_670_, DATT4) were strongly correlated with maize LCC values. However, RFR models with WV3 data had > 31% relative error, although the centre wavelength of the red band from WV3 data is 660.1 nm, which is the region absorbed by leaf chlorophyll a [63]. In comparison, RFR models with CUB data obtained slightly lower error, but all the essential WBs were between 682–702 nm (red-edge region) (Table 5 and Figure A2). This contrasts with results from another study using the same hyperspectral sensor (CUB) data, which reported the usefulness of WBs from blue, red, red-edge, and near-infrared regions for maize LCC estimation [64].

Indirectly linked VIs could estimate maize CWC in this study, while WI, which is a directly sensitive VI for CWC, showed the weakest relationship with CWC for all crops. This could be because crop parameters were highly correlated, and the variation of CWC somehow directly linked with the crop LAI and biomass values [25]. Nevertheless, water absorption at 970 nm due to O-H bonds in liquid canopy water [65] was one of the key WBs for maize CWC estimation by CUB data only (Table 5 and Figure A2).

### 4.4. Overall Discussion

This study could not conclude which RS data (spaceborne multispectral or UAV-borne hyperspectral) is better for the evaluated crop parameters for three crop types. Nevertheless, it is worth to mention the pros and cons of the two RS systems in terms of practical aspects of general crop monitoring. The spaceborne multispectral WV3 data hugely affected by cloud coverage in tropical regions, especially in the rainy season. Proper atmospheric corrections are needed to obtain accurate surface reflectance data from WV3 images to relate spectral values with crop vegetation parameters, which might not be easy to achieve. Additionally, the WV3 data cannot be acquired whenever it is needed because of its revisit frequency of one to five days, depending on the latitude. However, applying WV3 data to estimate crop parameter in the entire crop field can be efficiently performed because of the large spatial coverage of each satellite scene.

On the other hand, the UAV-borne CUB data can be collected whenever the data is needed, and there is no effect on the data due to cloud cover (when a proper radiometric correction is applied). However, coverage of a larger field needs to done using several UAV flight sessions, which could be a disadvantage over the WV3 data. Additionally, UAV-borne data is also challenging to collect in extreme weather conditions such as rain and wind, typical of the tropical region′s monsoon seasons.

**Figure A3:**
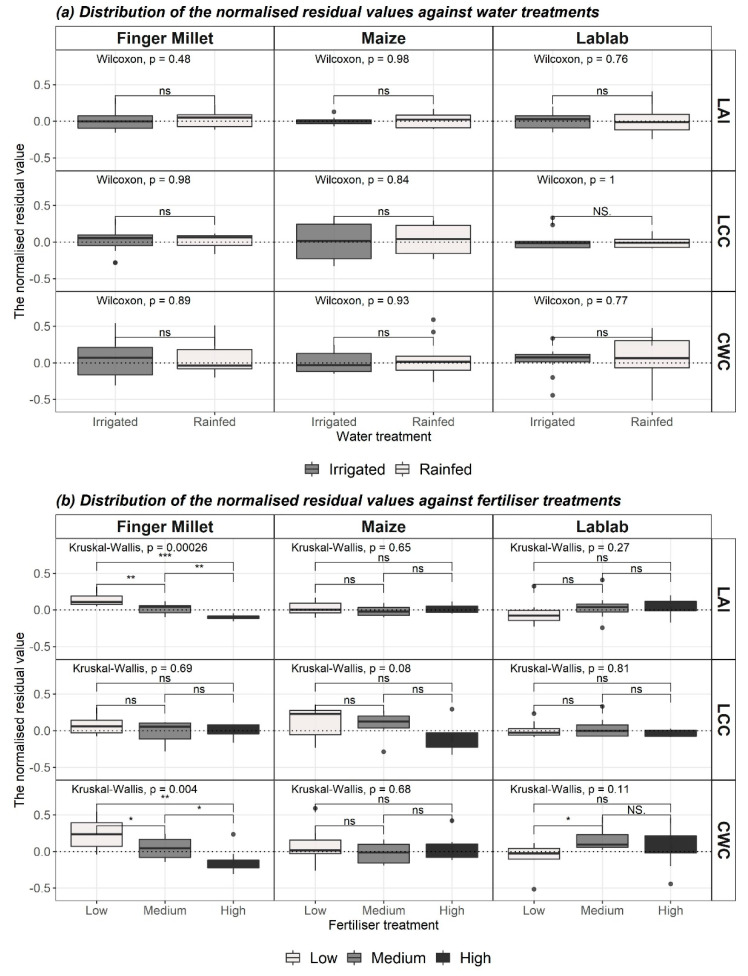
Distribution of the normalised residuals values against (**a**) water treatments and (**b**) fertiliser treatments from the best models for leaf area index (LAI) estimation, leaf chlorophyll content (LCC) estimation, and canopy water content (CWC) estimation for finger millet, lablab, and maize. The dashed line at y = 0 represents zero normalised residual value. (ns or NS: not significant, *: *p* < 0.05, **: *p* < 0.01, ***: *p* < 0.001).

This study’s third sub-objective explored how the crop parameter estimation accuracy was affected by the crop′s water and fertiliser treatments. The collected field data showed a significant positive effect due to irrigation in all three crops. However, finger millet (inflorescence emergence) and maize (development of fruit) were in similar phenological stages in both water treatments, while lablab showed two different phenological stages for irrigated and rainfed crops. (Table 1). The results clearly showed that the prediction accuracy of crop vegetation parameters did not significantly affect irrigation, and only finger millet′s LAI and CWC prediction error had a significant difference due to fertiliser treatments (Figure A3). Confirming these findings, [29] also reported no significant difference for biomass prediction error between two water treatments and fertiliser treatments for the same three crops with three-year data using in-situ hyperspectral data with machine learning methods.

This study utilised only a few (*n* = 24) samples for model building for vegetation parameter estimation. For this reason, separate models for the irrigation treatments were not employed, even though the data showed a significant difference between treatments. Therefore, the CV was applied to build unbiased models, which facilitated evaluating models with a limited number of data points from both treatments. However, the number of sample points for both training (*n* = 22) and validation (*n* = 2) in the CV was not enough to capture the dataset′s total variability. For example, when the model was trained with a unique range of dataset and the validation data points were out of the range from the trained model, then the model tends to under or overestimates the prediction value. It is necessary to have more data points to increase the model sensitivity to the dataset′s total variability. However, having many sample points is always challenging for RS-based crop parameter estimation for many reasons, including human and physical resource availability.

The two RS datasets used in this study were sensitive from the visible to the near-infrared region. According to published studies, usage of the spectral region until the shortwave infrared (2500 nm) could increase crop parameter estimation potential [12,24]. The two RS datasets utilised in this study could accurately estimate three crop vegetation parameters from three crop types with different agriculture treatments. Hence these results could be utilised as a starting point to an in-depth examination of how to use RS data without shortwave infrared spectral data for modelling LAI, LCC, and specifically CWC. Additionally, these research findings could be employed to monitor monsoon crops using the currently available spaceborne and UAV-borne high spatial resolution remote sensors with similar spectral sensitivity (e.g., Parrot Sequoia, Micasense RedEdge, and microsatellite constellations such as Planet).

## 5. Conclusions

This study focused on uncovering how two different spectral resolution RS data can be utilised for estimating crop vegetation parameters from three crops (finger millet, maize, and lablab) prominently grown in Southern India. This study evaluated two different very high spatial resolution (>1.5 m) RS spectral datasets (UAV-borne hyperspectral Cubert–CUB, spaceborne multispectral WorldView3–WV3) for estimating LAI, LCC, and CWC for the three target crops. Two distinct modelling methods, namely linear regression with best-correlated vegetation index and random forest regression with important wavebands, were also evaluated. According to the results, irrespective of the RS datatype, crop type, and modelling method, the average relative estimation error was less than 16%, 25%, and 22%, respectively, for LAI, LCC, and CWC estimation. However, there was no clear evidence to identify the best RS dataset or the best modelling method to estimate the examined crop parameters. Nevertheless, there was a trend that hyperspectral (CUB) data was better for estimation of vegetation parameters of finger millet while multispectral (WV3) data was better for both lablab and maize vegetation parameter estimation. Overall, vegetation indices derived from the combination of either green, red, red-edge, and near-infrared wavebands showed clear potential from either multi or hyperspectral data for an accurate estimation of the investigated vegetation parameters regardless of the crop type.

## Figures and Tables

**Table 1:** Phenological stages of the crops when the remote sensing and in-situ data were collected. Based on Table A3 from [29].

Crop	Phenological Stage (Days after Sowing)	
	Irrigated Experiment	Rainfed Experiment
**Finger millet**	Inflorescence emergence (87)	Inflorescence emergence (79)
**Lablab**	Ripening (83)	Development of fruit (78)
**Maize**	Development of fruit (87)	Development of fruit (79)

**Table 2:** WorldView-3 multispectral image′s bands and their effective bandwidths [32].

Band Name	Centre Wavelength (nm)	Effective Bandwidth (nm)
**Coastal blue (CB)**	427.4	40.5
**Blue (BL)**	481.9	54.0
**Green (GR)**	547.1	61.8
**Yellow (YE)**	604.3	38.1
**Red (RD)**	660.1	58.5
**Red-edge (RE)**	722.7	38.7
**Near-infrared 1 (N1)**	824.0	100.4
**Near-infrared 2 (N2)**	913.6	88.9

**Table 3:** Vegetation indices (VI) and their equations for WordView-3 (WV3) and Cubert (CUB) images. WV3 band names: GR: green, RD: red, RE: red-edge, N1: near-infrared 1. CUB bands are indicated by wavelength (*ρ*_xxx_) in nanometres. (NDVI: normalised difference vegetation index, DATT4: The 4th VI introduced by [36], MTVI: modified triangular vegetation index, REIP: red-edge inflexion point, and WI: water index)

VI	Formula for WV3 Bands	Formula for CUB Bands	Reference
**NDVI_800,670_**	$\frac{N1-RD}{N1+RD}$	$\frac{\rho_{800}-\rho_{670}}{\rho_{800}+\rho_{670}}$	[37]
**NDVI_750,550_**	$\frac{N1-GR}{N1+GR}$	$\frac{\rho_{750}-\rho_{550}}{\rho_{750}+\rho_{550}}$	[38]
**DATT4**	$\frac{RD}{GR\times RE}$	$\frac{\rho_{670}}{\rho_{550}\times \rho_{706}}$	[36]
**MTVI**	1.2 [1.2 (N1−GR)−2.5 (RD−GR)]	$1.2\;\left[1.2\;\left(\rho_{802}-\rho_{550}\right)-2.5\;\left(\rho_{670}-\rho_{550}\right)\right]$	[39]
**REIP**	$700+40\;\left[\frac{\left(\frac{RD+RE}{2}\right)-RE}{N1+RE}\right]$	$700+40\;\left[\frac{\left(\frac{\rho_{670}+\rho_{782}}{2}\right)-\rho_{702}}{\rho_{742}+\rho_{702}}\right]$	[40]
**WI**	$\frac{N1}{N2}$	$\frac{\rho_{902}}{\rho_{970}}$	[23]

**Table 4:** Summary of the crop parameter estimation model results from linear regression (LR) using the best-correlated vegetation index (VI). Bold values indicate the lowest nRMSEcv values among the two remote sensing datasets for each crop type. All the reported linear regression models with the best vegetation index showed *p*-value less than 0.05. (LAI: leaf area index, LCC: leaf chlorophyll content, CWC: canopy water content, CUB: Cubert, WV3: WorldView3, r: Pearson correlation coefficient between VI and crop-wise vegetation parameter, R^2^_cv_: coefficient of determination from cross-validation, nRMSE_cv_: normalised root means squares error from cross-validation).

Parameter	Crop	RS Data	LR Model with VIs			
			Best Vegetation Index	r	R^2^_cv_	nRMSE_cv_ (%)
**LAI (m^2^/m^2^)**	Finger millet	CUB	NDVI_800_670_	0.88	0.74	**15.7**
		WV3	REIP	0.88	0.74	16.1
	Lablab	CUB	NDVI_800_670_	0.90	0.77	**15.6**
		WV3	REIP	0.90	0.77	15.9
	Maize	CUB	NDVI_800_670_	0.90	0.77	**14.9**
		WV3	NDVI_800_670_	0.89	0.73	16.0
**LCC (µg/cm^2^)**	Finger millet	CUB	DATT4	0.83	0.63	**18.0**
		WV3	NDVI_750_550_	0.76	0.50	21.0
	Lablab	CUB	NDVI_750_550_	0.67	0.37	**23.3**
		WV3	NDVI_750_550_	0.66	0.36	23.4
	Maize	CUB	NDVI_800_670_	0.59	0.21	24.1
		WV3	DATT4	0.61	0.26	**23.3**
**CWC (kg/m^2^)**	Finger millet	CUB	NDVI_750_550_	0.73	0.44	**19.5**
		WV3	NDVI_750_550_	0.73	0.43	19.9
	Lablab	CUB	NDVI_800_670_	0.77	0.53	16.3
		WV3	REIP	0.81	0.58	**15.6**
	Maize	CUB	NDVI_800_670_	0.68	0.36	19.9
		WV3	DATT4	0.76	0.51	**17.0**

**Table 5:** Selected wavebands from Boruta feature selection algorithms for each crop vegetation parameter (LAI: leaf area index, LCC: leaf chlorophyll content, CWC: canopy water content) from two remote sensing datasets. Cubert bands are indicated as the band wavelength (*ρ*_xxx_) in nanometres.

**Parameter**	**Crop**	**Selected Wavebands from Cubert Data**	**Selected Wavebands from WorldView3 Data**
**LAI (m^2^/m^2^)**	Finger Millet	ρ_522_, ρ_526_, ρ_582_, ρ_642_, ρ_694_, ρ_702_, ρ_706_, ρ_722_, ρ_730_, ρ_738_, ρ_750_, ρ_762_, ρ_946_	Blue, Green, Yellow, Red, Red-edge, Near-infrared 2
	Lablab	ρ_690_, ρ_698_, ρ_706_, ρ_722_, ρ_726_, ρ_734_, ρ_750_, ρ_826_, ρ_918_, ρ_930_, ρ_946_, ρ_950_, ρ_954_, ρ_958_	Blue, Green, Yellow, Red, Red edge
	Maize	ρ_474_, ρ_478_, ρ_674_, ρ_682_, ρ_690_, ρ_694_, ρ_794_, ρ_802_, ρ_806_, ρ_822_, ρ_870_, ρ_874_, ρ_890_, ρ_898_, ρ_906_, ρ_930_, ρ_954_	Blue, Green, Yellow, Red, Red edge, Near-infrared 2
**LCC (µg/cm^2^)**	Finger Millet	ρ_746_, ρ_750_, ρ_754_, ρ_758_, ρ_762_, ρ_766_	Blue, Green, Yellow, Red, Red edge, Near-infrared 1, Near-infrared 2
	Lablab	ρ_574_, ρ_638_, ρ_718_, ρ_742_, ρ_750_	Blue, Green, Yellow, Red, Red edge
	Maize	ρ_682_, ρ_690_, ρ_698_, ρ_702_	Blue, Green, Yellow, Red, Red edge
**CWC (kg/m^2^)**	Finger Millet	ρ_470_, ρ_478_, ρ_522_, ρ_526_, ρ_694_, ρ_706_, ρ_710_, ρ_722_, ρ_742_, ρ_746_	Blue, Green, Yellow, Red, Red edge, Near-infrared 2
	Lablab	ρ_502_, ρ_606_, ρ_614_, ρ_618_, ρ_630_, ρ_666_, ρ_678_, ρ_682_, ρ_742_, ρ_802_, ρ_834_	Blue, Green, Yellow, Red, Red edge
	Maize	ρ_866_, ρ_878_, ρ_886_, ρ_918,_ ρ_966_, ρ_970_	Blue, Green, Yellow, Red, Red edge

**Parameter**

**Crop**

**Selected Wavebands from Cubert Data**

**Selected Wavebands from WorldView3 Data**

**Table 6:** Summary of the crop parameter estimation model results from random forest regression (RFR) using selected wavebands. Bold values indicate the lowest nRMSEcv values among the two remote sensing datasets for each crop type. (LAI: leaf area index, LCC: leaf chlorophyll content, CWC: canopy water content, CUB: Cubert, WV3: WorldView3, R^2^_cv_: coefficient of determination from cross-validation, and nRMSE_cv_: normalised root means squares error from cross-validation).

Parameter	Crop	RS Data	RFR Model with Selected Wavebands		
			No. of Wavebands	R^2^_cv_	nRMSE_cv_ (%)
**LAI (m^2^/m^2^)**	Finger millet	CUB	13	0.74	**16.1**
		WV3	6	0.70	17.1
	Lablab	CUB	14	0.84	12.9
		WV3	5	0.87	**12.0**
	Maize	CUB	18	0.79	13.9
		WV3	6	0.80	**13.9**
**LCC (µg/cm^2^)**	Finger millet	CUB	6	0.45	22.1
		WV3	7	0.51	**20.8**
	Lablab	CUB	5	0.23	**25.8**
		WV3	5	0.13	27.4
	Maize	CUB	4	0.16	**24.9**
		WV3	5	0.01	31.5
**CWC (kg/m^2^)**	Finger millet	CUB	10	0.43	**19.9**
		WV3	6	0.23	22.9
	Lablab	CUB	11	0.51	**16.9**
		WV3	5	0.42	18.2
	Maize	CUB	4	0.24	21.4
		WV3	5	0.26	**21.4**

## Data Availability

Not applicable.

## Appendix A

**Table A1:** Summary of the crop parameter data (LAI: leaf area index, LCC = leaf chlorophyll content, CWC: canopy water content, SD: standard deviation, CV: coefficient of variation).

Crop	Water	Min	Mean	SD	Max	CV
**LAI (m^2^/m^2^)**						
**Finger millet**	Irrigated	1.4	2.6	0.5	3.2	19.2%
	Rainfed	0.4	1.0	0.4	1.6	40.0%
**Lablab**	Irrigated	1.7	2.5	0.6	3.5	24.0%
	Rainfed	0.2	0.5	0.2	0.7	40.0%
**Maize**	Irrigated	2.1	2.7	0.2	3.0	7.4%
	Rainfed	1.0	1.6	0.4	2.2	25.0%
**LCC (µg/cm^2^)**						
**Finger millet**	Irrigated	19.3	39.7	13.6	65.6	34.3%
	Rainfed	10.2	12.8	3.4	21.4	26.6%
**Lablab**	Irrigated	17.5	36.3	7.8	43.0	21.5%
	Rainfed	20.1	27.5	4.3	33.3	15.6%
**Maize**	Irrigated	15.7	42.1	19.6	76.4	46.6%
	Rainfed	11.9	20.3	5.3	30.6	26.1%
**CWC (kg/m^2^)**						
**Finger millet**	Irrigated	0.4	1.4	0.7	2.7	46.5%
	Rainfed	0.1	0.5	0.2	1.0	52.1%
**Lablab**	Irrigated	0.4	0.7	0.3	1.6	48.5%
	Rainfed	0.03	0.08	0.04	0.1	50.0%
**Maize**	Irrigated	0.8	1.5	0.4	2.3	26.6%
	Rainfed	0.2	0.9	0.4	1.5	45.5%
